# Genome-scale analysis to the impact of gene deletion on the metabolism of *E. coli*: constraint-based simulation approach

**DOI:** 10.1186/1471-2105-10-S1-S62

**Published:** 2009-01-30

**Authors:** Zixiang Xu, Xiao Sun, Shihai Yu

**Affiliations:** 1 State Key Laboratory of Bioelectronics, Southeast University, Nanjing 210096, PR China; 2 Institute of science, PLA University of Science and Technology, Nanjing 211101, PR China

## Abstract

**Background:**

Genome-scale models of metabolism have only been analyzed with the constraint-based modelling philosophy. Some gene deletion studies on *in silico *organism models at genome-scale have been made, but most of them were from the aspects of distinguishing lethal and non-lethal genes or growth rate. The impact of gene deletion on flux redistribution, the functions and characters of key genes, and the performance of different reactions in entire gene deletion still lack research.

**Results:**

Three main researches have been done into the metabolism of *E. coli *in gene deletion. The first work was about finding key genes and subsystems: First, by calculating the deletion impact *p *of whole 1261 genes, one by one, on the metabolic flux redistribution of *E. coli*_iAF1260, we can find that *p *is more detailed in describing the change of organism's metabolism. Next, we sought out 195 important (high-*p*) genes, and they are more than essential genes (growth rate *f *becomes zero if deleting). So we speculated that under some circumstances and when an important gene is deleted, a big change in the metabolic system of *E. coli *has taken place and *E. coli *may use other reaction ways to strive to live. Further, by determining the functional subsystems to which 195 key genes belong, we found that their distribution to subsystems was not even and most of them were related to just three subsystems and that all of the 8 important but not essential genes appear just in "Oxidative Phosphorylation". Our second work was about *p*'s three characters: We analyzed the correlation between *p *and *d *(connection degree of one gene) and the correlation between *p *and *v*_*gene *_(flux sum controlled by one gene), and found that both of them are not of linear correlation, but the correlation between *p *and *f *is of highly linear correlation. The third work was about highly-affected reactions: We found 16 reactions with more than 2000 *R*g value (measuring the impact that a reaction is gotten in the whole 1261 gene deletion). We speculated that highly-affected reactions involve in the metabolism of basic biomasses.

**Conclusion:**

To sum up, these results we obtained have biological significances and our researches will shed new light on the future researches.

## Background

Since various 'omics' datasets are becoming available, biology has transited from a data-poor to a data-rich environment. This has underscored the need for systems analysis in biology and systems biology has become a rapidly growing field as well [1].

A change in mathematical modelling philosophy is also necessitated, and that is based on building and validating *in silico *models. Modern biological models need to meet new sets of criteria: organism-specific, data-driven, easily scalable, and so on. Many modelling approaches, such as kinetic, stochastic and cybernetic approaches, are currently being used to model cellular processes. Owing to the computational complexity and the large number of parameters needed, it is currently difficult to use these methods to model genome-scale networks. To date, genome-scale models of metabolism have only been analyzed with the constraint-based modelling philosophy [2,3]. Genome-scale network models of diverse cellular processes such as signal transduction, transcriptional regulation and metabolism have been generated. Gene-protein-reaction (GPR) associated models can keep track of associations between genes, proteins, and reactions [4], and there have been several genome-scale GPR models, such as *E. coli *[4,5], *S. aureus *[6], *H. pylori *[7], *M. barkeri *[8], *S. cerevisiae *[9] and *B. subtilis *[10]. A reconstruction is herein defined as the list of biochemical reactions occurring in a particular cellular system and the associations between these reactions and relevant proteins, transcripts and genes [2]. A reconstruction can include the assumptions necessary for computational simulation, such as maximum reaction rates and nutrient uptake rates [11].

Computer simulations of complex biological systems become essential as soon as the computational capability become available. As reconstructed networks have been made publicly available, researchers around the world have undertaken new computational studies using these networks [12]. Many researches apply a core set of basic *in silico *methods and often also describe novel methods to investigate different models. An extensive set of methods for analyzing these genome-scale models have been developed and have been applied to study a growing number of biological problems [12]. But as we have mentioned above, as yet, genome-scale models of metabolism have only been analyzed with the constraint-based philosophy [2,3].

The *in silico *models can be applied to generate novel, testable and often quantitative predictions of cellular behaviors [13]. The impact of a gene deletion experiment on cellular behavior can be simulated in a manner similar to linear optimization of growth [14]. The results can be used to guide the design of informative confirmation experiments and will be helpful for metabolic engineering. Some gene deletion studies on the genome-scale *in silico *models of organisms have been made [4-10,15-19], but most of them are from the standpoints of distinguishing lethal and non-lethal genes or growth rate [4-10,15-22]. The impact of gene deletion on flux redistribution, the characters and functions of key genes, and the performance of different reactions in entire gene deletion still lack research.

In this paper, in the part of results, we have done three research works. The first one: First, we calculated flux distribution of *E. coli*_iAF1260. Then we calculated the deletion impact of whole 1261 genes (using *p *to describe the deletion impact of one gene), one by one, on the metabolic flux redistribution of *E. coli*_iAF1260. Next, we sought out the important genes that most greatly affect the metabolic flux distribution, and furthermore determined their functional subsystems. The second one: We analyzed the correlation between *p *(describing deletion impact of one gene) and *f *(describing growth rate in the deletion of 1261 genes), the correlation between *p *and *d *(connection degree of one gene) and the correlation between *p *and *v*_*gene *_(flux sum controlled by one gene). The third one: We made research into what are the reactions affected most greatly in the whole 1261 gene deletion (using *R*g to measure the impact). In the part of methods and materials, we introduced the GPR model, some properties of the *in silico *model of *E. coli*_iAF1260 (SBML (Systems Biology Markup Language) format) and the method of constraint-based analysis.

## Results and discussion

### Metabolic flux distribution of *E. coli*_ iAF 1260

**Figure 1:**
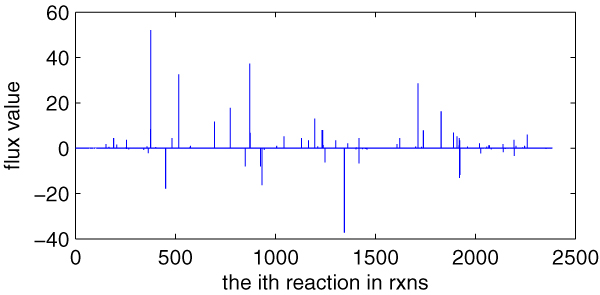
**Flux distribution of *E. coli*_iAF1260**. *X*-axis indicating every reaction in **rxns **(the order is as the same as in **rxns**, total 2382) and *y*-axis indicating the value of its corresponding flux (unit is mmol gDW^-1^h^-1^). **Rxns **is the reaction set in the model.

As a base for the later comparing research, we here calculate the flux distribution of *E. coli*_iAF1260. What we use is *E. coli*_iAF1260_ flux1.xml, one of the two SBML files that are presented with the reconstruction of *E. coli *[5]. The computational method we use is flux balance analysis (FBA) [11], one of the fundamental genome-scale phenotypic calculations, which can simulate cellular growth. FBA is based on linear optimization of an objective function, which typically is biomass formation. Given an uptake rate for key nutrients and the biomass composition of the cell (usually in mmol component gDW^-1 ^and defined in the biomass objective function), the maximum possible growth rate of the cells can be predicted *in silico*. We use the COBRA toolbox [11] to carry out this computation of FBA. The flux distribution of *E. coli*_iAF1260 is illustrated in Figure 1.

### Impact of gene deletion on the metabolic flux redistribution and key genes

As our first work, we now do research into the impact of gene deletion on the metabolic system of *E. coli*. First we calculate the deletion impact of 1261 genes, further seek out important genes and functional subsystems to which these key genes respectively belong.

#### 1) Impact of gene deletion on the metabolic flux redistribution and key genes that affect metabolism most greatly

There are 1261 genes in the model of *E. coli*_iAF1260. If a single gene is associated with multiple reactions, the deletion of that gene will result in the removal of all associated reactions. On the other hand, a reaction that can be catalyzed by multiple non-interacting gene products will not be removed in a single gene deletion. By the aid of the COBRA toolbox [11], we can calculate the impact of their deletion. We define the impact of one gene deletion on the whole metabolic flux redistribution as *p*

(1) $$p=\sum_{i}^{R}{\left({v^{\prime }}_{i}-v_{i}\right)}^{2}$$

Where *v*_*i *_and ${v^{\prime }}_{i}$ are respectively the flux value of *i*-th reaction of the model of *E. coli*_iAF1260 before and after a single gene deleting and *R *is the whole reaction set. In most of the researches on gene deletion [4-10,15-22], the change of growth rate *f *is often used to describe the impact of gene deletion. The reason why we define *p *as the impact of gene deletion is that we believe it is more detailed in describing the change of organism's metabolism. *p *has considered the flux change taking place at every reaction, and it uses the square sum of the difference between *v*_*i *_and ${v^{\prime }}_{i}$. Otherwise, *f *is just a whole measure and it does not distinguish the flux change taking place at every reaction.

**Figure 2:**
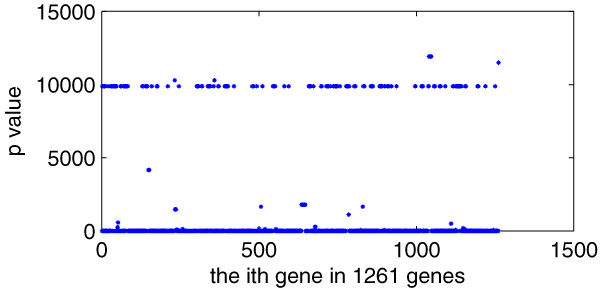
**The deletion impact *p *of 1261 genes of the *E. coli*_iAF1260 model**. *X*-axis indicating every gene in 1261 genes (the order is as the same as in **genes**, total 1261) and *y*-axis indicating its impact *p*. **Genes **is the set of genes in model.

Figure 2 shows the deletion impact of these 1261 genes. Table 1 gives *p *scopes, gene numbers falling within these scopes and their corresponding percentages that these genes take.

**Table 1:** *p *scopes, gene number (GN) and percentages

*p *scope	0	0–100	100–1500
GN	498	532	17

%	≈39%	≈42%	≈1%

*p *scope	1500–9800	>9800	
GN	19	195	
%	≈2%	≈15%	

**Figure 3:**
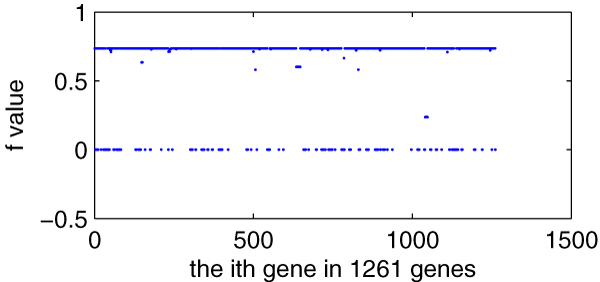
**The deletion impact of 1261 genes to *f *of the *E. coli*_iAF1260 model**. *X*-axis indicating every gene in 1261 genes and *y*-axis indicating new *f *after its deletion.

Figure 3 shows the deletion impact of these 1261 genes to the growth rate *f *of *E. coli*. Every deletion of these 1261 genes will entail a new *f*.

We define those genes with *p*>9800 as key genes or high-*p *genes, and there are 195 genes in total. There are 187 cases in which *f *= 0, their corresponding genes are usually called essential genes or zero-*f *genes, and all of their *p *are >9800. These 187 so-called essential genes are consistent with previous literatures [5], except "s0001" which is not included in the report of Ref. [5]. The left 8 genes with *p *> 9800 &*f *≠ 0 are shown in Table 2 with bold text, and we call them INE (Important but Not Essential) genes. Additional file 1 provides the details. Comparing with experiment observation [22], six (b3731, b3733, b3734, b3735, b3736, b3738, b3731) of the 8 INE genes are essential genes; Comparing with experiment observation [23], two (b3731, b3736) of the 8 INE genes are essential genes. At the same time, two genes (b0529 and b3956) are reported as essential genes in Ref. [5], but they are not key genes as for our computation, while b3956 is reported as nonessential gene both in Ref. [22,23] and b0529 is reported as nonessential gene both in Ref. [22]. From these comparisons, we can find that *p *has an advantage over *f *in describing the change of organism's metabolism.

**Table 2:** The functional subsystems (SS) and their related genes of *E. coli*_iAF1260

**SS**	**TLM**	**CPGB**	**VLIM**	**CEB**	**LBR**
**genes**	b0003	b0004, b0025, b0029	b0071	b0085	b0096
	b0004	b0052, b0103, b0109	b0072	b0086	b0179
	b0031	b0131, b0133, b0134	b0073	b0087	b0181
	b0166	b0142, b0154, b0159	b0074	b0088	b0182
	b2472	b0173, b0174, b0369	b3770	b0090	b0524
	b2478	b0414, b0415, b0417	b3771	b0091	b0914
	b2838	b0420, b0421, b0423	b3774	b0954	b0915
	b3359	b0475, b0750, b0907		b1093	b0918
	b3433	b1096, b1208, b1210		b1094	b1094
	b3809	b1277, b1662, b1740		b1288	b1215
	s0001	b1812, b2103, b2153		b2323	b3198
		b2315, b2320, b2400		b3176	b3633
		b2515, b2530, b2564		b3189	
		b2574, b2615, b2746		b3729	
		b2747, b2763, b2764		b3730	
		b2927, b3041, b3058		b3967	
		b3177, b3187, b3360		b3972	
		b3368, b3634, b3639			
		b3804, b3805, b3850			
		b3974, b3990, b3991			
		b3992, b3993, b3994			
		b3997, b4039, b4040			
		b4407, s0001			

**SS**	**MM**	**APM**	**GM**	**MLM**	**PPB**

**genes**	b0159	b0159	b0175	b0185	b0522, b0523
	b2687	b0386	b2585	b1092	b0945, b1062
	b2942	b2818	b3018	b1094	b1131, b1281
	b3939	b3172	b4041	b2316	b2312, b2476
	b4013	b3957	b4160	b2323	b2499, b2507
	s0001	b3958		b3255	b2557, b2780
		b3959		b3256	b3642, b4005
		b3960			b4006, b4177
		s0001			b4244, b4245

**SS**	**NSP**	**CAC**	**GSM**	**TTPM**	**TIM**

**genes**	b0639	b0720	b0907	b0908, b1260	b0914
	b1098	b1136		b1261, b1262	s0001
	b2827			b1263, b1264	
	b3648			b1693, b2329	
	s0001			b2599, b2600	
				b3389	

**SS**	**AAM**	**FM**	**ACM**	**HM**	**CM**

**genes**	b0928	b1415	b1415	b2019, b2020	b2750, b2751
		b3941	b3608	b2021, b2022	b2752, b2762
				b2023, b2024	b2763, b2764
				b2025, b2026	b3607

**SS**	**IITM**	**OP**	**U**		

**genes**	b3040	**b3731, b3732**	s0001		
	b3196	**b3733, b3734**			
	s0001	**b3735, b3736**			
		**b3737, b3738**			

* genes (text in bold) are important but not essential genes

We also note that there are 8 genes with *p*>9800 &*f *≠ 0. Based on the fact, we can speculate that, under some circumstances and when an important gene is deleted, a big change in the metabolic system of *E. coli *has taken place and *E. coli *may use other reaction ways to strive to live. This may reflect the robustness of the metabolic networks of microbes. It is also an important and interesting conclusion.

#### 2) Functional subsystems to which these key genes belong

If a gene catalyzes a reaction which belongs to a certain subsystem, we say that the gene belongs to the subsystem. Functional subsystems about important genes in the metabolic system of micro-organism are seldom reported. We have hereinabove defined those genes with *p*>9800 as key genes. We now list the functional subsystems to which every key gene belongs, 23 subsystems in total, and several genes appear in more than one subsystem, shown in Table 2. The 23 functional subsystems are "Threonine and Lysine Metabolism (TLM), Cofactor and Prosthetic Group Biosynthesis (CPGB), Valine Leucine and Isoleucine Metabolism (VLIM), Cell Envelope Biosynthesis (CEB), Lipopolysaccharide Biosynthesis Recycling (LBR), Methionine Metabolism (MM), Arginine and Proline Metabolism (APM), Glycerophospholipid Metabolism (GM), Membrane Lipid Metabolism (MLM), Purine and Pyrimidine Biosynthesis (PPB), Nucleotide Salvage Pathway (NSP), Citric Acid Cycle (CAC), Glycine and Serine Metabolism (GSM), Tyrosine Tryptophan and Phenylalanine Metabolism (TTPM), Transport Inner Membrane (TIM), Alanine and Aspartate Metabolism (AAM), Folate Metabolism (FM), Alternate Carbon Metabolism (ACM), Histidine Metabolism (HM), Cysteine Metabolism (CM), Inorganic Ion Transport and Metabolism (IITM), Oxidative Phosphorylation (OP), Unassigned (U)".

We can find that the distribution to subsystems of these 195 key genes is not even and most of them are related to "Cofactor and Prosthetic Group Biosynthesis", "Cell Envelope Biosynthesis" and "Purine and Pyrimidine Biosynthesis" subsystems, especially CPGB. We can also find that all of the important but not essential (INE) genes, 8 in total, appear in "Oxidative Phosphorylation".

The reason for many high-*p *genes just belonging to several metabolic subsystems maybe is in that these subsystems involve many reactions and provide supports for other subsystems; The reason for INE genes just belonging to "Oxidative Phosphorylation (OP)" subsystem probably is in that the permissibility which *E. coli *use other reaction ways to carry out this kind of metabolism, under the given media condition, takes place on OP subsystem.

### Analysis to the three characters of *p*

As our second work, we now begin research into some properties of the metabolic network of *E. coli*, i.e., three characters of *p*. Some properties about the metabolic network of micro-organisms have been reported in literatures [15-22]. Because the measure we defined is different, our research will provide further evidences to the properties about the metabolic network.

#### 1) Correlation between *p *and *f *(describing growth rate in the deletion of 1261 genes)

**Figure 4:**
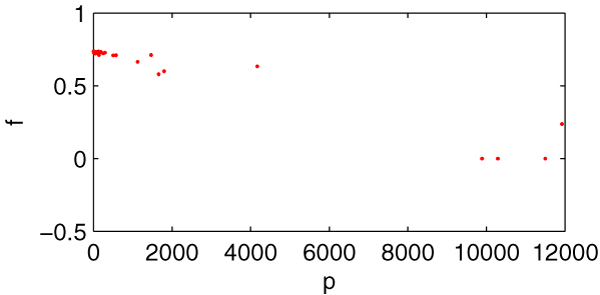
**The scatter diagram (*p*, *f*)**. *X*-axis indicating *p *and *y*-axis indicating *f*, total 1261 data pairs. Many data pairs locate at the same points.

Figure 4 is the scatter diagram (*p*, *f*), total 1261 data pairs. Many data pairs are superposition and locate at the same place, so there aren't lots of points in the figure. From the diagram, we can easily find that the relationship between *p *and *f *is of highly linear correlation. High *p *corresponds to low *f*.

#### 2) Correlation between *p *and *d *(connection degree of every gene in network)

**Figure 5:**
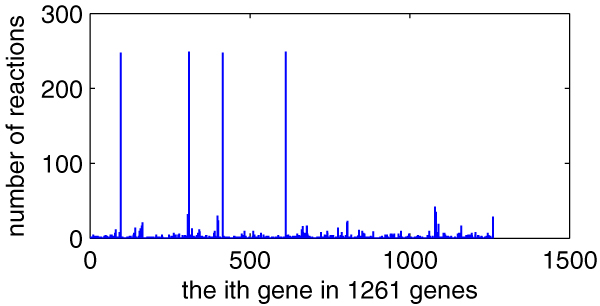
**The related reaction number of every gene in 1261 genes of the *E. coli*_iAF1260 model**. *X*-axis indicating every gene in 1261 genes (the order is as the same as in **genes**, total 1261) and *y*-axis indicating the number of its related reactions.

We compute out the related reaction number *d *of every gene in those 1261 genes of the *E. coli*_iAF1260 model, as illustrated in Figure 5. From the figure, we can find that some but not many genes have high *d *value, but we don't know whether they affect metabolic flux distribution greatly.

**Figure 6:**
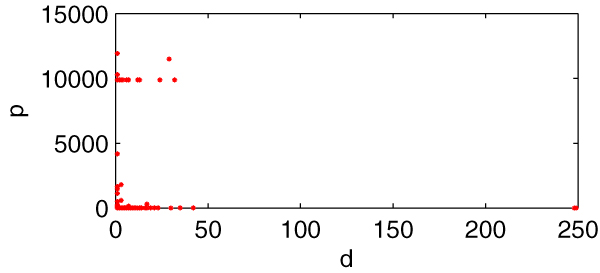
**The scatter diagram (*d*, *p*)**. *X*-axis indicating *d *(connection degree of every gene) and *y*-axis indicating the corresponding gene impact *p*.

Figure 6 is the scatter diagram (*d*, *p*), 1261 data pairs in total. Still many data pairs are superposition and locate at the same place. From the diagram, we can easily find that the relationship between *d *and *p *is not of linear correlation. So high-*d *genes and low-*d *genes are equally important to the metabolism of *E. coli*_iAF1260.

#### 3) Correlation between *p *and *v*_*gene *_(flux sum controlled by every gene)

We define the flux sum controlled by every gene as

(2) $$v_{gene}=\sum_{j}^{R_{gene}}\left|v_{j}\right|$$

**Figure 7:**
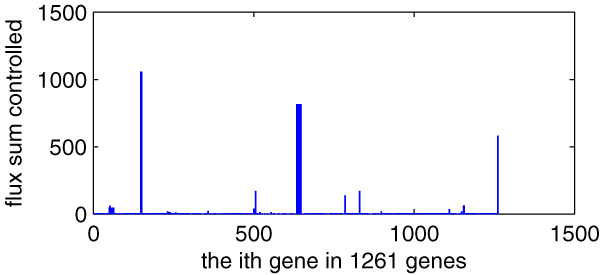
**The controlled reaction number of every gene in 1261 genes of the *E. coli*_iAF1260 model**. *X*-axis indicating every gene in 1261 genes (the order is as the same as in **genes**, total 1261) and *y*-axis indicating the number of its controlled reactions.

Where *v*_*j *_is the flux value of *j*-th reaction of the model of *E. coli*_iAF1260 before a single gene deleting and *R*_*gene *_is the reaction set controlled by the given gene. We can easily compute out the flux sum *v*_*gene *_of every gene in those 1261 genes of the *E. coli*_iAF1260 model, as illustrated in Figure 7. From the figure, we can find that some but not many genes have high *v*_*gene *_value, but will they affect metabolic flux distribution greatly?

**Figure 8:**
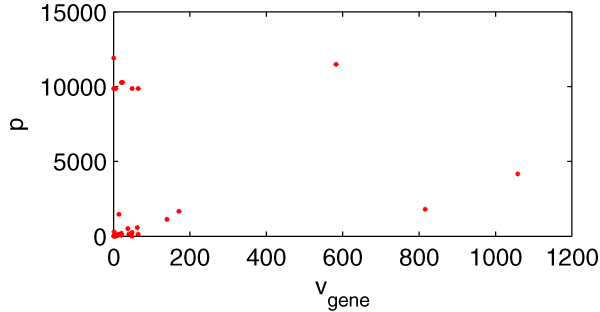
**The scatter diagram (*v*_*gene*_, *p*)**. *X*-axis indicating *v*_*gene *_(the flux sum controlled by every gene) and *y*-axis indicating the impact, *p*.

Figure 8 is the scatter diagram (*v*_*gene*_, *p*), 1261 data pairs in total, and many data pairs are superposition. From the diagram, we can also find that the relationship between *v*_*gene *_and *p *is not of linear correlation as well. So genes with high *v*_*gene *_and genes with low *v*_*gene *_are equally important to the metabolism of *E. coli*_iAF1260.

### Impact of gene deletion on every metabolic reaction

As our third work, we now make research into what are the reactions affected most greatly in the whole 1261 gene deletion. Highly-affected reactions (HAR) are often neglected in many researches in literatures about gene deletion study.

#### 1) Impact of gene deletion on every metabolic reaction

There are 2382 reactions in the *in silico *model of *E. coli*_iAF1260. We define *R*g to measure the impact that a reaction is gotten in the whole 1261 gene deletion.

(3) $$R_{g}=\sum_{k}^{G}\left|v_{k}-v_{0}\right|$$

Where *v*_0 _and *v*_*k *_are respectively the flux value of a certain reaction of the model of *E. coli*_iAF1260 before and after *k*-th gene deleting, and *G *is the set of whole 1261 genes.

**Figure 9:**
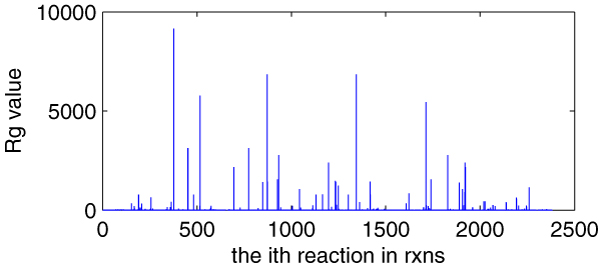
**The *R*g of each 2382 reactions of *E. coli*_iAF1260**. *X*-axis indicating every reaction in 2382 reactions (the order is as the same as in **rxns**, total 2382) and *y*-axis indicating its corresponding *R*g value.

Figure 9 provides each *R*g of 2382 reactions and Table 3 shows *Rg *scopes, corresponding reaction number within these scopes and the percentages that these reactions take. In the following section, we will determine what the highly-affected reactions are.

**Table 3:** *R*g scopes, reaction number (RN) and their percentages

*Rg *scopes	0	0–20	20–60
RN	876	1279	114

%	≅37%	≅54%	≅5%

*Rg *scopes	60–500	>500	

RN	71	42	

%	≅3%	≅2%	

#### 2) Highly-affected reactions (HAR)

There are 42 reactions which the *R*g value of every one of them is beyond 500. Especially, for those with more than 2000 *R*g value, there are 16 reactions in total, and they are "ATPS4rpp, CO2tex, CO2tpp, CYTBO3_4pp, ENO, EX_co2(e), EX_h2o(e), EX_ o2(e), GAPD, H2Otex, H2Otpp, NADH16pp, O2tex, O2tpp, PGK, PGM".

Why are these 16 reactions more sensitive to gene deletion? Maybe, it is due to the fact that they involve in the metabolism of basic biomasses such as H_2_O, ATP, O_2_, NADH.

## Conclusion

In this paper, we have done three main researches into the metabolism of *E. coli *in gene deletion. The first was to find its important genes and the corresponding belonging subsystems, the second was to analyze the characters of *p*, and the third was to find its highly-affected reactions in gene deletion.

To the first work: We used *p *to describe the impact which gene deletion entailed. Our first finding was that maybe *p *is more detailed than *f *in describing the change of organism's metabolism in gene deletion. After calculating the deletion impact of 1261 genes, we sought out 195 important genes (high *p *genes, *p *>9800), and they are more than essential genes (*f *= 0 genes). So our second finding was that under some circumstances and when an important gene is deleted, the metabolic system of *E. coli *has greatly changed and *E. coli *may use other reaction ways to strive to live. The third finding was that the distribution to subsystems of these 195 key genes is not even and most of them are related to about three subsystems ("Cofactor and Prosthetic Group Biosynthesis", "Cell Envelope Biosynthesis" and "Purine and Pyrimidine Biosynthesis") and that all of the 8 important but not essential (INE) genes appear just in "Oxidative Phosphorylation" subsystem. We have also tried to give some explanations.

To the second work: We have done research into *p*'s three characters, i.e. its relationship with *f*, *d*, *v*_*gene*_. We found that *p*-*f *correlation was of highly linear correlation, while both of the *p*-*d *correlation and the *p*-*v*_*gene *_correlation were not of linear correlation. Our research can provide further evidences to the properties about the metabolic network, because the measure we defined is different.

To the third work: We defined *R*g to measure the impact that a reaction is gotten in the whole 1261 gene deletion. We calculated the *R*g value of each 2382 reactions and gave a statistics to the *Rg *scopes and the corresponding reaction number. Finally, we sought out 16 reactions with more than 2000 Rg value. We have also tried to give an explanation, i.e., these highly-affected reactions involve in the metabolism of basic biomasses.

In summary, because the *in silico *model of *E. coli*_iAF1260 is credible, we can conclude that the results we obtained have biological significances and that the researches we have done will shed new light on the future research. As a next step, we will try more media conditions to the research on *E. coli*, and will also do similar work on other organisms and compare them with the case of *E. coli*.

## Methods

### Gene-protein-reaction (GPR) associated model

The association between genes and reactions is not a one-to-one relationship. Many genes may encode subunits of a protein which catalyze one reaction, while there are genes that encode so-called promiscuous enzymes that can catalyze several different reactions. So it is necessary to keep track of associations between genes, proteins, and reactions and to distinguish "&" and "OR" associations in GPR models. Examples of different types of GPR associations are illustrated in Ref. [4,14].

### GPR model structure of *E. coli*_iAF1260

The *in silico *model that we use is *E. coli*_iAF1260 [5], a metabolic reconstruction consisting of the chemical reactions that transport and interconvert metabolites within *E. coli *K-12 MG1655. This network reconstruction was based on a previous reconstruction, termed *E. coli*_iJR904 [4]. The general features of *E. coli*_iAF1260 are shown in Ref. [5].

SBML format file to the model *E. coli*_iAF1260 can be downloaded from the supplementary information of Ref. [5]. There are two SBML files that are presented with the reconstruction, each containing a different flux distribution XML files. SBML file properties are given in the supplementary of Ref. [5]. The dimensions of **rxns**, **mets**, and **genes **are respectively 2382, 1668, 1261.

The minimal media of *in silico *model is an important aspect. The computational minimal media of *E. coli*_iAF1260 is also included in the supplementary information of Ref. [5]. In the method of constraint-based analysis, the biomass objective function (BOF) should be defined. The BOF was generated by defining all of the major and essential constituents that make up the cellular biomass content of *E. coli *[5].

Gene-protein-reaction associations embodied in **rxnGeneMat **matrix, which is a matrix with as many rows as there are reactions in the model and as many columns as there are genes in the model. The *i*th row and *j*th column contains a one if the *j*th gene in **genes **is associated with the *i*th reaction in **rxns **and zero otherwise.

### Methodology of constraint-based analysis

#### 1) Constraint-based analysis

*In silico *modelling and simulation of genome-scale biological systems are different from that practiced in the physicochemical sciences. A network can fundamentally have many different states or many different solutions. Which states (or solutions) are picked is up to the cell and based on the selection pressure experienced, and such choices can change over time. Therefore, constraint-based approaches [2,3] to the analysis of complex biological systems have proven to be very useful. The differences between the physicochemical sciences and the physical sciences or engineering are illustrated in Ref. [14]. All theory-based considerations (i.e., engineering and physics) lead one to attempt to seek an "exact" solution, and typically computed based on the laws of physics and chemistry. However, constraint-based considerations (as in biology) are useful. Not only can a network have many different behaviors that are picked based on the evolutionary history of the organism, but also these networks can carry out the same function in many different and equivalent ways [14].

#### 2) Representation of reconstructed metabolic network

Before calculation and simulation, the reconstructed metabolic network must be represented mathematically. The stoichiometric matrix, ***S***, is the centerpiece of a mathematical representation of genome-scale metabolic networks. It represents each reaction as a column and each metabolite as a row, where each numerical element is the corresponding stoichiometric coefficient.

An upper and lower bound for the allowable flux through each reaction also requires defining. This represents the lowest and highest reaction rate possible for each reaction. The set of upper and lower bounds is represented as two separate vectors, each containing as many components as there are columns in ***S***, and in the same order. In many cases, reversible reactions are defined to have an arbitrary large upper bound and an arbitrarily large negative lower bound. Irreversible reactions have a lower bound that is nonnegative, usually zero.

In order to predict meaningful fluxes, setting upper and lower bounds is especially important for exchange reactions which serve to uptake compounds to the cell or secrete compounds from the cell. The lower bound of exchange reaction column must be a finite negative number using this orientation (e.g., glucose). The upper bound of exchange reaction column must be greater than zero. At least one of the reactions in the model must have a constrained lower/upper bound, and typically, the substrate (e.g., glucose or oxygen) uptake rates are set to experimentally measured values. The upper and lower bounds for exchange reactions are quantitative *in silico *representations of the growth media environment.

#### 3) Biomass objective function (BOF) and minimal media

The constraint-based approach is based on the assumption that cells strive to maximize their growth rate. This assumption which provides an acceptable starting point for many types of computations is satisfied by simulating maximal production of the molecules required to make new cells (biomass precursor molecules). In spite of their limitations, the predictive power of genome-scale models of metabolic networks has been demonstrated in diverse situations through careful experimentation [11].

The biomass objective function (the function *v*_*growth*_, see below) is a special reaction taking as substrates of all biomass metabolites, ATP and water and producing ADP, protons, and phosphate (as a result of the non-growth associated ATP maintenance requirement) [6].

The minimal media is determined computationally with the systematic testing of distinct inputs. Different combinations of molecules are allowed to enter the reaction network until the minimal group that allowed biomass production, or non-zero *Z *(see below), was found [6]. It is only concerned that some amount of biomass production is calculated but do not discriminate between extremely slow, inefficient growth and rapid growth.

#### 4) Computation of phenotypic states

In genome-scale metabolic networks, the fluxes within a cell usually cannot be uniquely calculated because a range of feasible values exist when fluxes are subjected to known constraints. Flux balance analysis (FBA) is used to find optimal growth phenotypes. Briefly, a large-scale linear programming is used to find a complete set of metabolic fluxes (***v***) that are consistent with steady-state condition (eq. 4) and reaction rate bounds (eq. 5), and at the same time maximize the biomass objective function in the defined ratio. This corresponds to the following linear programming problem [6]:

max *Z *= *v*_*growth*_

Subject to

***S***·***v ***= **0**

*α*_*i *_<*v*_*i *_<*β*_*i*_

Where ***S ***is the stoichiometric matrix, and *α*_*i*_and *β*_*i *_define the bounds through each reaction *v*_*i*_. The flux range was set arbitrarily high for all internal reactions so that no internal reaction restricted the network, with the exception of irreversible reactions, which have a minimum flux of zero. The inputs to the system were restricted to a minimal media.

The value of *Z *computed with the above procedure can either be zero (predicting no growth) or greater than zero (corresponding to cellular growth) depending on the inputs and outputs that are allowed, according to the nutrients provided in the media.

#### 5) Gene deletion study

The effect of a gene deletion experiment on cellular growth can be simulated in a manner similar to linear optimization of growth [5,11]. Gene-reaction associations model the logical relationship between genes and their corresponding reactions. If a single gene is associated with multiple reactions, the deletion of that gene will result in the removal of all associated reactions, i.e. to simultaneously restrict the fluxes (upper and lower flux bounds) of these reactions to zero prior to computing maximal biomass objective function. On the other hand, a reaction that can be catalyzed by multiple non-interacting gene products will not be removed in a single gene deletion. The possible results from a simulation of a single gene deletion are unchanged maximal growth (non-lethal), reduced maximal growth or no growth (lethal). Those genes were considered essential if no biomass could be produced without their usage.

## List of abbreviations

GPR: Gene-protein-reaction; SBML: Systems Biology Markup Language; FBA: Flux balance analysis; INE: Important but Not Essential; HAR: Highly-affected reactions; BOF: Biomass objective function; TLM: Threonine and Lysine Metabolism; CPGB: Cofactor and Prosthetic Group Biosynthesis; VLIM: Valine Leucine and Isoleucine Metabolism; CEB: Cell Envelope Biosynthesis; LBR: Lipopolysaccharide Biosynthesis Recycling; MM: Methionine Metabolism; APM: Arginine and Proline Metabolism; GM: Glycerophospholipid Metabolism; MLM: Membrane Lipid Metabolism; PPB: Purine and Pyrimidine Biosynthesis; NSP: Nucleotide Salvage Pathway; CAC: Citric Acid Cycle; GSM: Glycine and Serine Metabolism; TTPM: Tyrosine Tryptophan and Phenylalanine Metabolism; TIM: Transport Inner Membrane; AAM: Alanine and Aspartate Metabolism; FM: Folate Metabolism; ACM: Alternate Carbon Metabolism; HM: Histidine Metabolism; CM: Cysteine Metabolism; IITM: Inorganic Ion Transport and Metabolism; OP: Oxidative Phosphorylation; U: Unassigned

## Competing interests

The authors declare that they have no competing interests.

## Authors' contributions

ZX collected the data, carried out the computation, performed the analysis, and drafted the manuscript. XS and SY participated in the design of the study. All authors read and approved the final manuscript.

## Supplementary Material

Additional file 1 **The detailed comparison**. The file (detailed_comparison.xls) includes two contents: The first is the comparison between those 188 essential genes reported in Ref. [5] and those 195 important genes that we obtained from computation. The second is the comparison between *p *and *f *of every gene deletion that we obtained from computation. Click here for file
